# Transferance of Measles to Dogs

**Published:** 1876-09

**Authors:** 


Transferance of Measles to Dog.—Dr. Squire recently narrated to the London Epidemological Society an instance of the conveyance of measles from man to the dog. A dog licked the hands of a child in bed with the rash of measels at its hight. The dog sickened on the twelfth day, suffered from coryza two days, and died on the fourth day of illness, with most characteristic congestion of the throat and air-passage. The dog had gone through distemper four years before; a proof that the usual form of distemper in dogs is not measles.—*Medical Times.*

